# Population Structure and Genetic Diversity of the *Toona ciliata* (Meliaceae) Complex Assayed with Chloroplast DNA Markers

**DOI:** 10.3390/genes15030320

**Published:** 2024-02-28

**Authors:** Zi-Yun Wang, Ying Hu, Yan-Wen Lv, Yu Xiao, Zi-Han He, Chao Wu, Xin-Sheng Hu

**Affiliations:** 1 College of Forestry and Landscape Architecture, South China Agricultural University, Guangzhou 510642, China; iamwang_ziyun@163.com (Z.-Y.W.); yinghu2023@163.com (Y.H.); yanwenlvyx@126.com (Y.-W.L.); yxiaoyu06@163.com (Y.X.); hezhan1717@163.com (Z.-H.H.); chaowu1998@163.com (C.W.); 2 Guangdong Key Laboratory for Innovative Development and Utilization of Forest Plant Germplasm, Guangzhou 510642, China

**Keywords:** *Toona*, haplotype diversity, chloroplast marker, isolation by distance, genetic conservation

## Abstract

*Toona ciliata* is a deciduous or semi-deciduous tree species and belongs to the *Toona* genus of the Meliaceae family. Owing to low natural regeneration and over-exploitation, the species is listed as an endangered species at level II in China and its conservation has received increasing concern. Here, we sampled 447 individuals from 29 populations across the range-wide distribution of the *T. ciliata* complex in China and assessed their genetic variation using two chloroplast DNA markers. The results showed that the overall haplotype diversity and nucleotide diversity per site were high at *h* = 0.9767 and π = 0.0303 for the *psb*A-*trn*H fragment and h= 0.8999 and π = 0.0189 for the *trn*L-*trn*L fragment. Phylogenetic analysis supported the division of the natural distribution of *T. ciliata* complex into western and eastern regions. The genetic diversity was higher in the western region than in the eastern region, showing significant phylogeographic structure. Genetic differentiation among populations was moderate ($\Phi_{st}=42.87\%$), and the effects of isolation by distance (IBD) were significant. A neutrality test and mismatch distribution analysis indicated that the distribution of the *T. ciliata* complex generally did not expand, although a few local populations could likely expand after bottleneck effects. The overall results were complementary to and consolidated previous studies using mitochondrial and nuclear DNA markers. We finally discussed strategies for the genetic conservation of the *T. ciliata* complex.

## 1. Introduction

*Toona* is a genus of deciduous or semi-deciduous broad-leaved trees in the Meliaceae family and is mainly distributed in subtropical and tropical areas [1]. The genus includes four species in China, including *T*. *sinensis*, *T. ciliata*, *T. macrocarpa*, and *T. rubriflora*, among which *T. sinensis* and *T. ciliata* are the most extensively exploited. *T. ciliata* is widely distributed in southern China, covering Yunnan, Guangxi, Guizhou, Sichuan, Hunan, Hubei, Fujian, and Guangdong Provinces. Based on the phenotypic variation in leaf and floral traits, five varieties of *T. ciliata* are recognized, including *T. ciliata* var. *ciliata*, *T. ciliata* var. *yunnanensis*, *T. ciliata* var. *pubescens*, *T. ciliata* var. *sublaxiflora*, and *T. ciliata* var. *henryi* [1]. These varieties are geographically distributed in sympatry or parapatry and grow in hills and mountains at elevations between 300 m and 2260 m above sea level. Natural hybridization among varieties cannot be excluded, although a few reports on their hybrids are available in the literature. Because leaf and floral traits are often influenced by environmental factors and exhibit continuous variation within and between species [2,3], the delimitation of varieties of *T. ciliata* based on these traits remains uncertain. There is still debate about the taxonomic status of these varieties [4]. Similar to a previous study that did not specify a variety, here we bring these five varieties together as the *T. ciliata* complex. Individual samples of *T. ciliata* were not identified to any specific variety.

*T. ciliata* is an important fast-growing timber species and is also known as the Chinese mahogany [5,6,7]. One remarkable feature is that *T. ciliata* has a tough texture, straight grain, beautiful pattern, dark reddish-brown heartwood, and lighter sapwood, and hence, it has a significant timber value [8,9]. The species also has a potential medicinal value since extracts from its branches and leaves have various biological activities, including antibacterial, antiviral, molluscacide, and antimalarial effects [9]. The species is considered as one of the preferred species to adjust the species structure of coniferous and broad-leaved mixed forests in the hills of southern China [9]. It is of great significance for developing forest production to vigorously promote the development of *T. ciliata*. However, due to its low natural regeneration [10] and over-exploitation, *T. ciliata* is recognized as an endangered species at level Ⅱ, which is defined as an endangered species of important economic, cultural, or scientific research value. Therefore, its genetic conservation has received increasing concern.

Previous studies on the *T. ciliata* complex have mainly focused on cultivation, silviculture techniques, ecophysiology, timber properties, and forest community succession [11]. For instance, the mixed forests of *T. ciliata* with *Cunninghamia lanceolata*, *Pinus massoniana,* or *Pinus yunnanensis* could effectively prevent land decline caused by continuous planting, and improve soil fertility [12,13]. Genetic studies have focused on provenance trials [14,15], tissue culture [16], and cuttings propagation [17]. Population genetic studies with molecular markers include the investigation of genetic diversity and population structure of the *T. ciliata* complex using SRAP (sequence-related amplified polymorphisms) markers [18], ISSR (inter-simple sequence repeats) markers [19], SSR (simple sequence repeat) markers [20], and nuclear ITS (nuclear internal transcribed spacer) and mitochondrial DNA (mtDNA) markers [4]. They showed that there were significant population differentiation and isolation-by-distance (IBD) effects in the natural distribution of the *T. ciliata* complex in China. Xiao et al. [21] showed that four varieties of *T. ciliata* (without *T. ciliata* var. *sublaxiflora* among the five varieties) in sympatry were genetically well mixed in terms of chloroplast genomes. Other studies using SSR markers include the population genetic structure of *T. ciliata* var. *pubescens* [22,23]. Analysis of the mating system scored by nuclear SSR markers indicated that the *T. ciliata* complex is predominantly outcrossing, with partial selfing and inbreeding [24]. Recent genome sequencing provides a useful reference for studying the phylogenetic relationship among varieties and for developing nuclear molecular markers for breeding [25,26]. All these studies provide a general picture of the genetic diversity and population structure of the *T. ciliata* complex, which serves as a valuable reference to the genetic conservation of this endangered species in China.

The purpose of this study was to use chloroplast DNA (cpDNA) markers to investigate the population genetic diversity and population structure of the *T. ciliata* complex in China. This study is complementary to previous studies using mtDNA and unclear SRAP and ITS markers [4,18]. It is well known that chloroplast DNA markers have multiple attributes that are suitable for investigating genetic variation within and between species [27,28], including (i) uniparental inheritance (maternal inheritance for angiosperms and paternal inheritance for gymnosperms), (ii) a higher mutation rate per site compared with the plant mitochondrial genome but a lower mutation rate per site compared with the plant nuclear genome, and (iii) a small genome size (120–220 kb) [29]. These characteristics ensure that the genetic diversity derived from cpDNA markers is different from that derived from mtDNA and nuclear DNA markers. Moreover, gene flow for the cpDNA markers of angiosperms is mediated by seed flow only, similar to mtDNA markers but different from nuclear markers whose gene flow is mediated by both seed and pollen flow. It is expected in theory that population genetic differentiation derived from cpDNA markers is comparable to that from mtDNA markers if mutation effects are negligible between them [30]. Also, it is expected that genetic differentiation derived from cpDNA markers is greater than that from nuclear markers due to smaller genetic drift effects and both seed and pollen flow for the nuclear markers [31]. To compare with previous studies [18,21], we investigated range-wide populations of the *T. ciliata* complex. By synthesizing information on genetic variation from nuclear and organelle genome markers, we assessed the genetic diversity and population structure of the *T. ciliata* complex from the whole genome perspective. The overall results were then applied in discussing the genetic conservation strategy for the *T. ciliata* complex.

## 2. Materials and Methods

### 2.1. Sampling and DNA Extraction

The experimental sampling sites covered 11 provinces, and four hundred and forty-seven individuals of 29 populations were collected across the range-wide distribution of the *T. ciliata* complex in China (Table 1). Here, populations in different localities were determined according to China’s administrative division. These sampling sites were the same as those used by Li et al. [18] and Xiao et al. [4] but fewer samples were analyzed. Most leaf samples were collected from 2015 to 2016 [18]. Because some leaf samples were used in previous experiments [18], we collected some supplementary samples from provenance trials established at the Zengcheng Experiment Station (113°37′ E, 23°14′ N, and 20.3 m above sea level) and the Yuejin North Experiment Station, South China Agricultural University (113°37′ E, 23°16′ N, and 42.3 m above sea level), in 2018. Note that some populations only had a few samples because supplementary samples were not obtained. All sample trees were separated by at least 50 m. The altitudes of the sampling sites ranged from 337 m (Guanshan, Jiangxi Province) to 1862 m (Huidong, Sichuan Province), with an average altitude of 808.2 m.

Approximately 5 g of fresh leaves per sample were immediately dried and preserved on silica gel. Most DNA samples extracted from healthy leaves were provided by Li et al. [18]. Supplementary DNA samples were extracted from the leaves of living plants according to the CTAB 2X protocol [32]. The quality of DNA extraction was checked by 0.8% (*w*/*v*) agarose gel electrophoresis. All quantified DNA samples were stored at −80 °C for subsequent polymerase chain reaction (PCR) amplification.

### 2.2. Primer Screening, Amplification, and Sequencing

According to the literature [33], four pairs of universal cpDNA primers were checked, namely, *psb*A-*trn*H, *trn*T-*trn*L, *trn*L-*trn*L, and *trn*L-*trn*F (Table 2). Among them, the intergenic region of *psb*A-*trn*H is one of the fastest evolutionary regions in the chloroplast genome, and the sequence has been widely used in genetic diversity analysis [34]. After checking the gel bands of the PCR amplifications (Figure S1), we selected two primer pairs, i.e., *psb*A-*trn*H and *trn*L-*trn*L, for sequencing analyses.

PCR amplification was carried out in a 25 μL reaction system, consisting of 1 μL of genomic DNA, 1 μL of forward and reverse primers, 12.5 μL of 2 × ES Taq Mastermix (Yongjin Biotechnology, Guangzhou, China), and 9.5 μL of ddH_2_O. The PCR procedure was carried out in a Dongsheng Thermal Cycler (EDC-810, Suzhou, China). The procedure of the thermal cycling system for both primer pairs was as follows: pre-denaturation for 4 min at 94 °C, followed by 35 cycles of 94 °C for 1 min, 55 °C for 1 min, 72 °C for 1 min, and finally, 72 °C extension for 10 min. The amplification products were stored in the refrigerator at 4 °C and then promptly sent to Sangon Biotech (Shanghai, China) for sequencing. The peak maps obtained from sequencing were viewed and detected by Chromatogram Explorer3.2 (Figure S2), which indicated that there were no cluttered peaks or strong base signals. The sequences with high quality and no mixed peak signals were used for downstream analyses.

### 2.3. Analysis of Genetic Diversity

The sequenced fragments from PCR amplifications were aligned using MEGA 7 [35], removing the parts with heterozygous interferences caused by unstable signals at the fronts and ends. Ultimately, we obtained two datasets of sequences for 29 populations, one for the *psb*A-*trn*H fragment and the other for the *trn*L-*trn*L fragment (see Table S1 in Supplementary Materials for concatenated alignment sequences). We used DnaSP v5 [36] to estimate haplotypes (see Tables S2 and S3 in Supplementary Materials for haplotype sequences), observed haplotype diversity (h), and nucleotide diversity (π) per site for each population and the overall samples. The observed haplotype diversity (*h*) for each population was estimated by

(1) $$h=1-\sum_{i}p_{i}^{2}$$

where $p_{i}$ is the frequency of the *i*th haplotype in the focal population [37]. Since many haplotypes were detected, instead of drawing a complex network, we used parsimony tree by MEGA 7 [35] to depict the evolutionary relationships among haplotypes.

### 2.4. Population Genetic Structure

We used DNAsp v5 to estimate genetic differentiation coefficients, including $N_{st}$ [38,39], $G_{st}$ [37], and $F_{st}$ [40]. We tested whether $N_{st}$ was larger than $G_{st}$ or not to infer if the phylogeographic structure occurred. Similarly, Arlequin v3.0 [41] was used for molecular analysis of variance (AMOVA) to calculate the distribution of genetic variation within and between populations and to estimate genetic differentiation $\Phi_{st}$ [42] between populations.

Isolation by distance (IBD) was tested using regression analysis of ${(F}_{st}/(1-F_{st}))$ on the logarithm of geographic distance [40,43]:

(2) $$\frac{F_{st}}{1-F_{st}}=a+b\cdot ln(geographicdistance).$$

A significant difference of the regression coefficient *b* from zero indicates the presence of IBD effects. Note that the geographical distance between pairwise populations was calculated using their longitude and latitude coordinates (Table 1). A Mantel’s test was also conducted to examine the relationship between $F_{st}$ and geographic distance [44]. Pearson’s correlation (r) between haplotype diversity and elevation was tested for each fragment.

Phylogenetic relationships among 447 individuals of 29 populations were constructed by MEGA 7 [35]. The phylogenetic tree among populations was also drawn using the ML (maximum likelihood) method [36]. In addition, we investigated the population structure by ADMIXTURE 1.3.0 using the concatenated sequences of the *psb*A-*trn*H and *trn*L-*trn*L fragments [45].

### 2.5. Population Demography

Tajima’s D [46] and Fu’s F [47] tests were conducted to indicate if a population had experienced expansion after bottleneck effects. Neutrality was tested using Arlequin v3.0 [41] with the two chloroplast DNA fragments. It is expected that both Tajima’s D and Fu’s F are negative if the population has expanded after bottleneck effects. In addition, a mismatch distribution was analyzed for each population. When the observed frequency of pairwise different sites has a single-peaked distribution, consistent with a single-peaked Poisson distribution, population expansion is implied after bottleneck effects. Statistical tests were performed using the sum of squares (SSD) and Harpending’s raggedness index (Rag) to check whether the expected SSD (or Rag) was greater than the observed SSD (or Rag). Estimates of other parameters included $\theta_{0}=2N_{1}\mu$ and $\theta_{1}=2N_{2}\mu$, where $N_{1}$ and $N_{2}$ are the population sizes before and after population expansion, respectively, and τ(t) is the time elapsed since the sudden expansion of the population. 

## 3. Results

### 3.1. Haplotype Analysis

**Figure 1:**
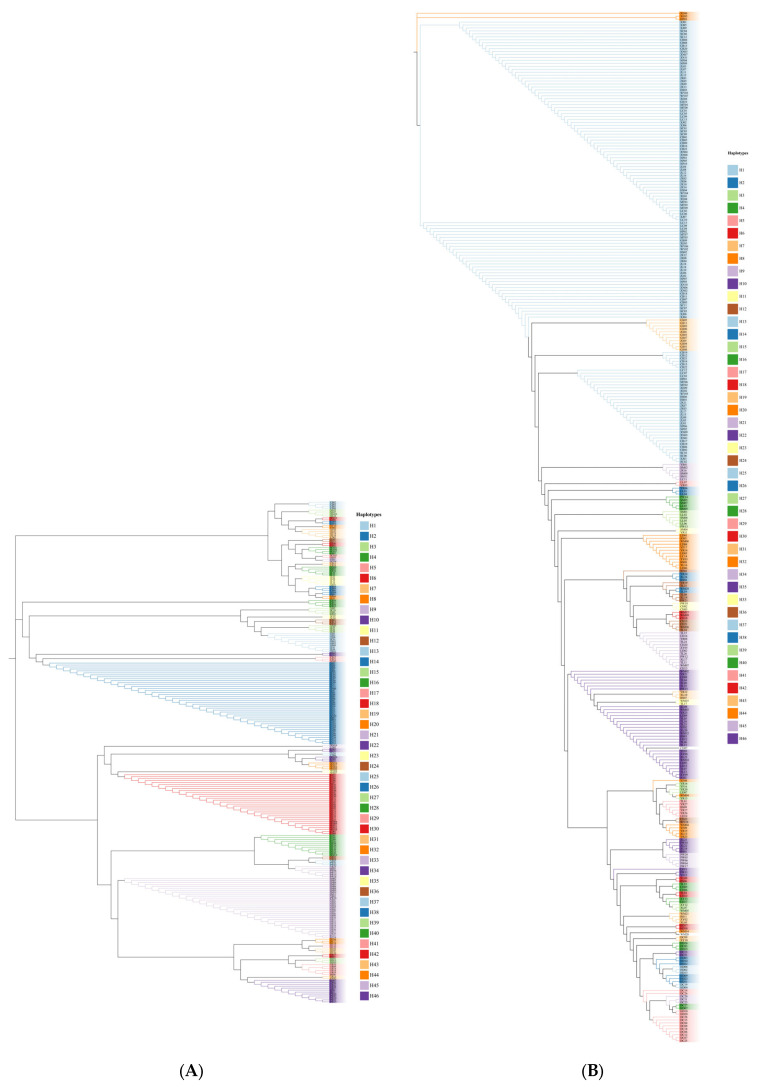
Phylogenetic relationships among haplotypes H1–H46: (**A**) *psb*A-*trn*H fragment and (**B**) *trn*L-*trn*L fragment. Note that the haplotype sequences for the same code Hi (i = 1, …, 46) were different between the two fragments.

The amplified sequences from the *psb*A-*trn*H fragment were about 564bp after alignment among 447 individuals. Analysis with DNAsp v5 identified 239 haplotypes among the 447 samples (Table 3). There were 193 haplotypes in total whose abundance was one in overall samples (447 individuals). For simplicity, we used code Hi (i = 1, …, 46) to represent haplotypes whose abundances were not less than two and code H47 to represent all haplotypes occurring only once in the overall samples. Populations DC, TL, CH, and HD possessed more than 20 haplotypes, while population YF was fixed by haplotype H9. Haplotype H2 was the most frequent haplotype, accounting for 9.4% of the overall samples and occurring in eight populations. Figure 1A shows the evolutionary relationship among the 46 haplotypes. Only a few haplotypes were dominant, and most haplotypes were derived from a few main haplotypes with a couple of mutant bases.

For the sequence of the *trn*L-*trn*L fragment, we identified 143 haplotypes from 447 samples (Table 4). There were also 46 haplotypes whose abundances were not less than two and denoted by Hi (i = 1, …, 46). There were 97 haplotypes that occurred only once in the overall samples. For simplicity, these single-abundant haplotypes were commonly denoted by code H47. Populations XL, WM, and YR possessed more than 20 haplotypes, while populations XJ, SC, NP, JL, and MT were fixed by haplotype H1. Haplotype H1 was the most frequent haplotype, accounting for 29.8% of the overall samples and occurring in 15 populations. Figure 1B shows that most single-abundant haplotypes were derived from a few domain haplotypes with a couple of mutations.

The analysis of haplotype diversity indicated that the observed haplotype diversity ranged from 0 (YF) to 0.9586 (TL) for the *psb*A-*trn*H fragment (Table 3). The mean of observed haplotype diversity across populations was 0.7391 ± 0.2477. The nucleotide diversity per site (π) ranged from 0 (YF) to 0.1398 (HS) with a mean of 0.0303 ± 0.0374 (Table 3). The diversity in the overall samples was *h* = 0.9767 and π = 0.0303.

For the *trn*L-*trnL* fragment (Table 4), the observed haplotype diversity (*h*) ranged from 0 to 0.9489 (WM) with a mean of 0.5556 ± 0.3739. Multiple populations (XJ, SC, XN, NP, JL, and MT) were fixed by haplotype H1. The nucleotide diversity per site (π) ranged from 0 to 0.0154 (JG) with a mean of 0.0044 ± 0.0039. The diversity in the overall samples was *h* = 0.8999 and π = 0.0189.

**Figure 2:**
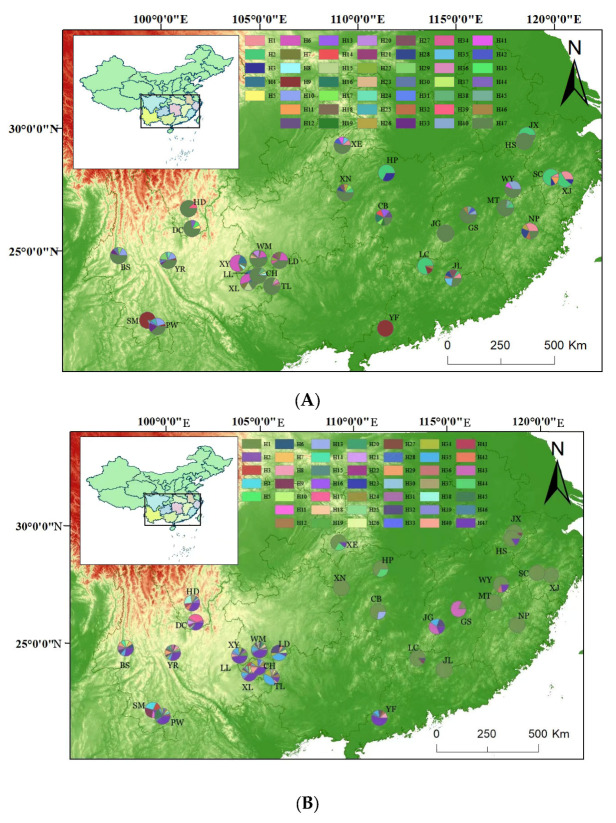
A map showing the twenty-nine sample sites and the geographic distribution of the cpDNA haplotypes of the *psb*A-*trn*H and *trn*L-*trn*L fragments: (**A**) the *psb*A-*trn*H fragment and (**B**) the *trn*L-*trn*L fragment. The pie charts show different proportions of haplotypes within each population. Different haplotypes are denoted by code Hi (i = 1, 2, … 47). Each color in the pie charts represents one haplotype (H1–H47).

Figure 2 shows the geographical distribution of haplotypes for the *psb*A-*trn*H fragment (A) and the *trn*L-*trnL* fragment (B). A generally similar pattern of diversity was observed for both fragments. Populations had larger haplotype diversity in the western region than in the eastern region. An explicit phylogeographic structure was present in the natural distribution of the *T. ciliata* complex.

### 3.2. Population Genetic Structure

For the *psb*A-*trn*H fragment, AMOVA showed that 26.55% of the total genetic variation was from among populations (*p*-value = 0.0000) and 73.45% was from within populations (Table 5). For the *trn*L-*trn*L fragment, AMOVA showed that a major proportion of genetic variation occurred among populations, accounting for 70.02% (*p*-value = 0.0000). For the concatenated sequences of the *psb*A-*trn*H and *trn*L-*trn*L fragments, AMOVA showed that 42.84% of the genetic variation occurred among populations (*p*-value = 0.0000) and 57.16% occurred from within populations. All these results indicated a significant level of difference in the genetic variation among populations of the *T. ciliata* complex.

**Figure 3:**
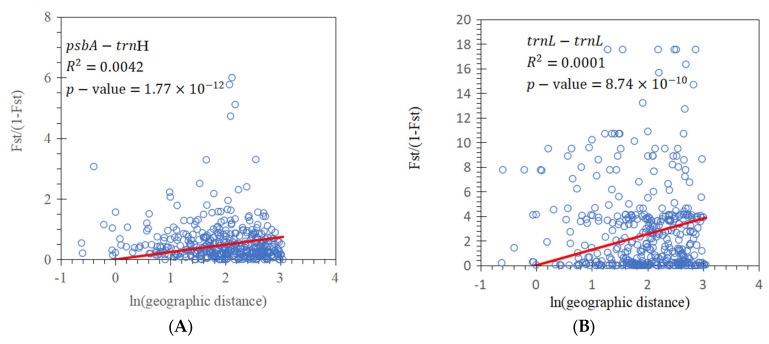
Tests of the effects of isolation by distance (IBD) on population genetic differentiation: (**A**) the *psb*A-*trn*H fragment and (**B**) the *trn*L-*trn*L fragment. Significant but weak IBD effects occurred among populations. The red line in each figure indicated the trend of the relationship between Fst/(1-Fst) and the logarithm of geographic distance.

A comparison of $N_{st}$ and $G_{st}$ confirmed the presence of phylogeographic structure in the distribution of haplotype diversity. Analysis with the *psb*A-*trn*H sequences showed that *G_st_* (=0.1546) was significantly smaller than $N_{st}$ (0.2194) (*p*-value < 5%). Analysis with the *trn*L-*trn*L sequences showed that $G_{st}$ (0.2531) was also significantly smaller than $N_{st}$ (0.6972). These results were consistent with the pattern of the haplotype diversity distribution (Figure 2). IBD tests indicated significant but small correlations between genetic differentiation and geographical distances (*psb*A-*trn*H: *R*^2^ = 0.0042, *p*-value = 2.02 × 10^−12^; *trn*L-*trn*L: *R*^2^ = 0.0001, *p*-value = 9.02 × 10^−10^) (Figure 3). Pearson’s correlation tests indicated that the observed haplotype diversity was not significantly correlated with elevation for the *psb*A-*trn*H fragment (r = 0.3528, *p*-value = 0.0605) but was significantly correlated with elevation for the *trn*L-*trn*L fragment (r = 0.4082, *p*-value = 0.0279).

**Figure 4:**
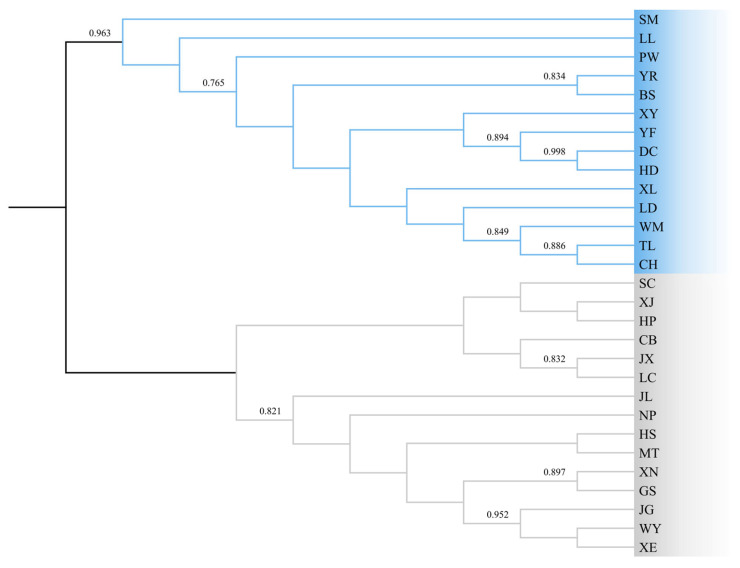
Phylogenetic relationships among 29 populations of the *T. ciliata* complex derived from concatenated sequences of the *psb*A-*trn*H and *trn*L-*trn*L fragments. The maximum likelihood method with 1000 bootstrap resamples was used to draw the consensus tree. Populations in grey and blue were grouped into eastern and western regions, respectively.

Figure 4 shows a consensus tree among 29 populations. Generally, the twenty-nine populations could be divided into two groups: the western and eastern regions. The western region covered Yunnan (SM, PW, BS, YR), Guizhou (XY, WM, CH, LD), Guangxi (TL, XL, LL), Sichuan (DC, HD), and Guangdong (YF, LC) Provinces. The eastern region covered Fujian (NP, WY), Jiangxi (MT, GS, JL, JG), Anhui (HS, JX), Hunan (XN, HP, CB), Zhejiang (SC, XJ), and Hubei (XE) Provinces. Generally, the two regions were connected to each other in the geographical distribution of the *T. ciliata* complex.

**Figure 5:**
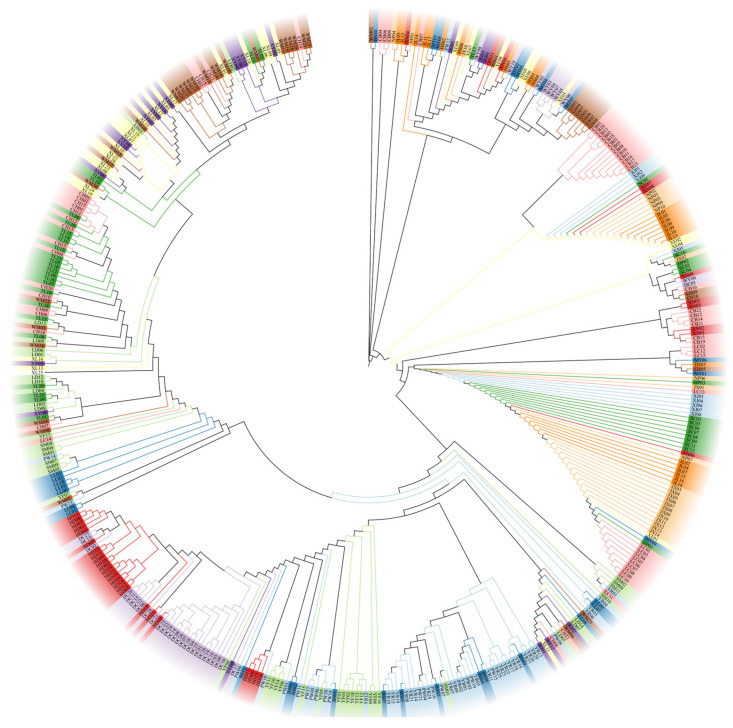
Phylogenetic relationships among 447 individuals using the concatenated sequences of the *psb*A-*trn*H and *trn*L-*trn*L fragments. The tree was drawn by the maximum likelihood method.

**Figure 6:**
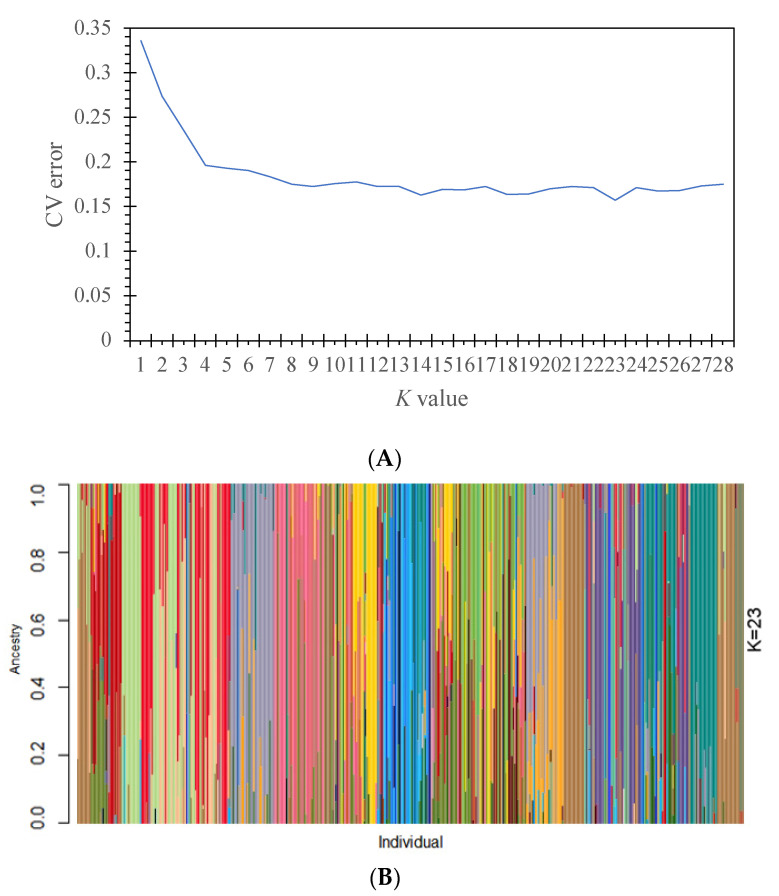
Population structure analysis of 447 individuals of the *T. ciliata* complex. (**A**) Cross-validation (CV) errors for the number of subpopulations (*K*) changing from 1 to 28. (**B**) A partitioned map of individuals with clustering assignments (*K* = 23) indicated in different colors. The proportions of genetic components from different subpopulations in each individual were indicated in different colors.

The analysis of genetic relationships showed a good genetic mixture among the 447 individuals (Figure 5). Many individuals from different populations were more genetically related. A further analysis with ADMIXTURE showed that the optimal number of subpopulations was *K* = 23, with the minimum cross-validation (CV) error (CV error = 0.1574) (Figure 6). Individuals across populations were genetically well mixed by different proportions of the optimal 23 populations, supporting the phylogenetic relationships among individuals (Figure 5).

### 3.3. Analysis of Population Demography

Neutrality tests with the concatenated sequences of the *psb*A-*trn*H and *trn*L-*trn*L fragments indicated that most populations had negative Tajima’s D or Fu’s F values (Table 6). Eight populations had significant Tajima’s D values (*p*-value < 0.05), including populations LL, SM, DC, WM, BS, TL, JX, and PW. Seven populations had significant Fu’s F values (*p*-value < 0.05), including populations DC, YR, LD, TL, CH, HD, and MT. Only two populations (DC and TL) exhibited significant Tajima’s D and Fu’s F values (negative). There were four populations with positive Tajima’s D values, including SC, CB, NP, and JG, suggesting that these populations had not experienced expansion.

**Figure 7:**
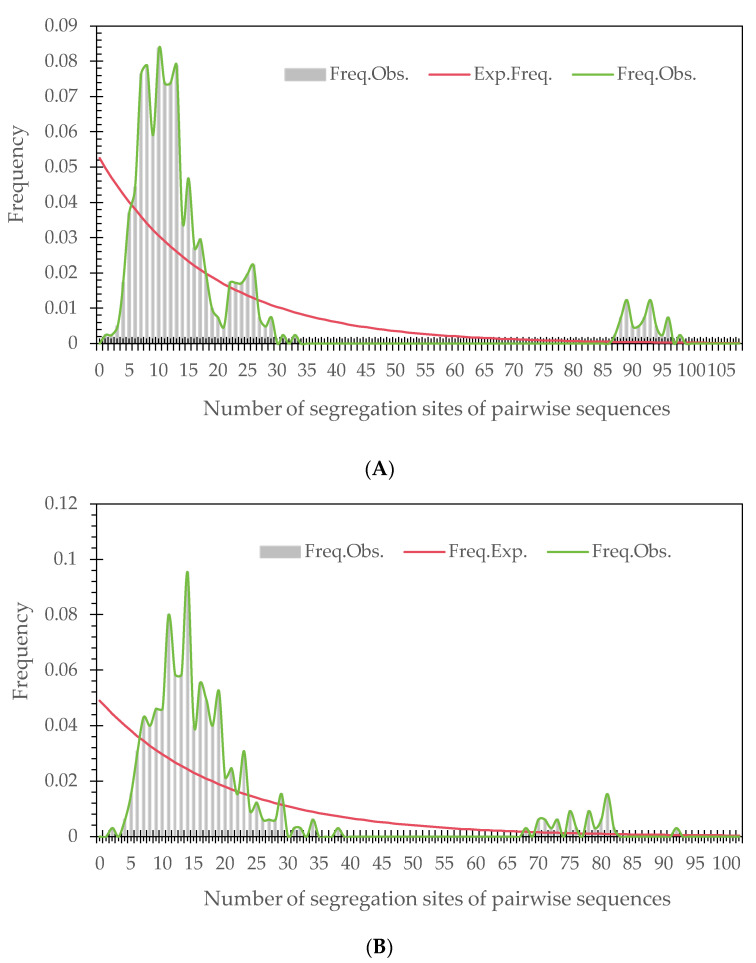
Analysis of mismatch distribution using the concatenated sequences of the *psb*A-*trn*H and *trn*L-*trn*L fragments: (**A**) population DC and (**B**) population TL. The grey bar lines and green lines are the observed frequencies under different numbers of segregation sites of pairwise sequences, whereas the red lines are the expected frequencies under a model of sudden population expansion [48].

Mismatch distribution analyses indicated that populations with significant Tajima’s D or Fu’s F did not expand significantly, with the probabilities of *P* (expected SSD ≥ observed SSD) < 0.95 and *P* (expected RAG ≥ observed RAG) < 0.95 (Table 6). The actual observed curves were essentially multimodal or sawtooth-shaped, indicating that these populations had not recently experienced expansion or a range expansion. Figure 7 shows the mismatch distributions of populations DC and TL with negative Tajima’s D and Fu’s F values, suggesting that these two populations did not expand after bottleneck effects. Taken together, populations of the *T. ciliata* complex generally did not experience expansion in its natural distribution in China, although a couple of local populations could exhibit potential expansion.

## 4. Discussion

### 4.1. Genetic Diversity

This study is complementary to previous studies that used both nuclear and mitochondrial DNA markers [4,18,20]. The results consolidate previous results of genetic diversity derived from SRAP, SSR, and nuclear ITS markers. Combining the results from organelle (mtDNA, cpDNA) and nuclear genome (SRAP, SSR, and ITS) markers, we conclude two common patterns regarding the spatial distribution of genetic diversity: (1) The range-wide distribution can be generally divided into western and eastern regions, although the boundaries between them are difficult to delineate. (2) The western region harbors larger genetic diversity than the eastern region. Note that the western region may be further divided into two regions based on mtDNA markers [4]. It is speculated that the *T. ciliata* complex could likely initialize in the western region and move forward to the eastern side of China, although the original center of *T. cilata* remains to be determined. This could likely have a similar origination to *T. sinensis* in China [49]. Lu et al. [49] thought that Chinese *T. sinensis* could originate from the regions of northeastern India and Burma. This needs further verification in future studies.

As expected, cpDNA markers had greater haplotype diversity and nucleotide diversity per site than mtDNA markers [4]. The haplotype diversity of these two cpDNA fragments in the overall samples (the observed *h* = 0.9767 for the *psb*A-*trn*H fragment and the observed *h* = 0.8999 for the *trn*L-*trn*L fragment) is also greater than some other plants, such as *Camellia huana* (*h* = 0.759) [50], *Michelia shiluensis* (*h* = 0.674) [51], *Iris loczyi* (*h* = 0.820) [52], *Rhododendron rex* (*h* = 0.788) [53], *Vouacapoua americana* (*h* = 0.87) [54], and *Tilia cordata* (*h* = 0.881) [55]. One main reason is the higher mutation rates occurring in these two fragments, although the general mutation rate over the whole chloroplast genome is not high [27]. Many unique haplotypes with a couple of mutations that are different from major haplotypes likely occurred from recent mutations (Figure 1), which enhances the overall haplotype and nucleotide diversity.

The distribution of haplotypes in the western region was consistent with previous results. Xiao et al. [21] showed well-mixed phylogenetic relationships among individuals of four varieties of *T. ciliata* in Yunnan Province (*T. ciliata* var. *ciliata*, *T. ciliata* var. *yunnanensis*, *T. ciliata* var. *pubescens*, and *T. ciliata* var. *henryi*), implicating close genetic relationships among individuals in the western region in terms of chloroplast genomes. The distribution of haplotypes in the entire region showed an obvious phylogeographic structure, and different haplotypes were not randomly distributed in space. For example, dominant haplotype H1, coded in both fragments, appeared only in the eastern region of the whole distribution, and each was distributed in geographically similar ranges. These consolidate the previous study showing a phylogeographic structure in the *T. ciliata* complex with nuclear ITS markers [4].

### 4.2. Population Genetic Structure

The analysis of genetic structure among all populations showed moderate and significant genetic differentiation, accounting for 42.84% of the total genetic variation. This level of differentiation is lower than that derived from haploid nuclear SRAP (79.2%) [18], mtDNA (88.84%), and nuclear ITS (71.43%) [4] but greater than the genetic differentiation derived from SSR markers with the same 29 populations (32.4%) [20] and with six different populations (34.5%) [24]. As expected, the genetic differentiation derived from cpDNA markers is greater than that of some tropical forest species derived from nuclear allozymes [56]. In theory, when gene flow and genetic drift are the only two processes under the neutral hypothesis, population differentiation is expected to be greater for cpDNA markers of maternal inheritance than for nuclear markers of biparental inheritance but comparable between cpDNA and mtDNA markers with maternal inheritance [31]. However, the preceding comparison is not in good agreement with this theoretical expectation, where genetic differentiation with cpDNA markers is smaller than that with ITS and mtDNA markers. 

One possible explanation is that high mutation rates in these two cpDNA fragments produced moderate genetic differentiation. This could be inferred in theory from Wright’s formula under equilibrium of drift–migration–mutation in island model [30]: $F_{st}={\left(1+2N_{e}(m_{s}+\mu )\right)}^{-1}$, where $m_{s}$ is the migration rate of seeds and μ is the mutation rate. A high mutation rate results in small genetic differentiation, which was supported from a comparison of *F_st_* values derived from the SSR versus ITS markers (32.4% vs. 71.43%) with the same populations [4,20]. The mutation rate of SSR markers is often considered to be higher than that of ITS markers. This could explain why the *F_st_* derived from cpDNA markers is smaller than that derived from mtDNA markers or potentially smaller than that derived from nuclear ITS markers despite lower genetic drift effects for nuclear genomes [31]. A comparison of genetic differentiation between the two markers (Table 5) implies that the mutation rate in the *psb*A-*trn*H fragment could be greater than that in the *trn*L-*trn*L fragment, as was discussed by Zhu et al. [34] in medicinal plants.

A further insight into the effects of high mutation rates on genetic differentiation could be approximated from a comparison of $F_{st(cp)}={\left(1+2N_{e}(m_{s}+\mu_{cp})\right)}^{-1}$ for cpDNA markers and $F_{st(mt)}={\left(1+2N_{e}(m_{s}+\mu_{mt})\right)}^{-1}$ for mtDNA markers under equilibrium. Assuming that the mutation rate for mtDNA markers is negligible ($\mu_{mt}\approx 0$), we can obtain the following expression:

(3) $$\frac{\mu_{cp}}{m_{s}}=\frac{\frac{1}{F_{st(cp)}}-1}{\frac{1}{F_{st(mt)}}-1}-1$$

Substitution of $F_{st(mt)}=0.8884$ [4] and $F_{st(cp)}$ = 0.4284 into Equation (3) yields a point estimate of $\mu_{cp}/m_{s}$ = 9.6234 ≫ 1.0, which is analogous to the genetic variation at copy number variation loci in *Homo sapiens* [57]. The mutation rates at these two fragments are likely not as small as we generally thought before, and they play an important role in shaping the population genetic structure at these two loci.

Population structure in woody species is affected by multiple ecological and life history traits, including taxonomic status (angiosperm versus gymnosperm), regional distribution, geographical range, mating system, and seed dispersal [58]. The geographical range of the *T. ciliata* complex involves complicated landscape structure (fragmented) and connectivity. From the results assayed with mtDNA markers [4], the investigated populations were divided into three groups from the elevations of 617.34 ± 264.19 m in the eastern region to 732.37 ± 250.87 m in the central region and 1407.67 ± 321.81 m in the western region. The central populations investigated (XY, LL, WM, XL, CH, TL, LD, and YF) were mainly located in Guizhou and Guangxi Provinces and showed small genetic variation among them due to geographic proximity. However, the overall range harbors diverse habitats that caused population isolation and intensified genetic differentiation among populations of the *T. ciliata* complex. Significant IBD effects could be an important factor causing substantial genetic differentiation at the range-wide scale, which was detected by both organelle and nuclear genome markers [4]. The result indicating correlations between haplotype diversity and elevation implies that mountain ranges played a certain role in blocking gene flow at the range-wide scale [4]. This is similar to the pattern of subtropical and tropical *Machilus pauhoi* in South China [59]. The terrain in the eastern region is mostly mountain, which hinders both pollen and seed dispersal, resulting in less obvious pollen flow than that in the western region, in contrast to the western region, where pollen flow is more extensive than seed flow [4].

In addition, *T. ciliata* is mainly pollinated by insects [7], which could also enhance population structure. A study on the mating system of *T. ciliata* showed that pollen spread within and between populations was limited in different areas of the species’ distribution [24]. This may partly arise from the over-exploitation that led to low population density or the limited number of pollen donors within or between populations. Predominantly outcrossing with partial selfing and inbreeding facilitates IBD effects and, hence, population differentiation. The phylogenetic relationship among populations was closely linked to the geographical location, indicating an explicit haplotype geographical structure.

### 4.3. Genetic Conservation

The neutrality tests implied that these two markers were essentially neutral in all populations except for DC and TL based on both Tajima’s D and Wu’s F tests. The whole demography of the *T. ciliata* complex generally did not expand, although a few local populations likely expanded after bottleneck effects. The analysis of mismatch distribution also supported no general expansion in population demography. Previous analyses of maxent models showed no obvious widespread expansion of *T. ciliata* in the whole geographic range, although the whole range of *T. ciliata* could appear with some local expansions to the north but contraction in the south [60,61,62]. Thus, there is no strong evidence to suggest that the natural distribution of the *T. ciliata* complex will substantially expand in the future [4].

Information on well-mixed genetic relationships among all samples derived from phylogenetic relationships (Figure 5 and Figure 6) is partially consistent with the results of four varieties assayed with chloroplast sequences [21]. This supports the previous recommendation of conserving the genetic variation of this species in terms of the *T. ciliata* complex rather than individual varieties. Moreover, the differentiation between western and eastern regions coupled with IBD effects implies that multiple populations should be considered in the genetic conservation of the *T. ciliata* complex. Xiao [4] suggested that only a few of the populations could be appropriate for conservation in the central region based on an analysis with mtDNA and nuclear ITS markers. This is also supported by the analysis with cpDNA markers in the central region (mainly in Guizhou and Guangxi Provinces). Current over-exploitation of land and anthropogenic disturbances have exacerbated geographical and genetic isolation, which further impedes *T. ciliata* regeneration and fragments the distribution of the *T. ciliata* complex in China. Therefore, in formulating strategies for the conservation of genetic resources, we need to consider the conservation of multiple populations in the western and eastern regions in addition to the traditional model of extensive development and breeding. As an endangered species at level II in China, it is necessary to strengthen the protection of the existing genetic variation in the *T. ciliata* complex.

## 5. Conclusions

*T. ciliata*, an endangered species at level II in China, has received increasing concern for its genetic conservation. Here, we used cpDNA markers to investigate the genetic diversity and population structure of the *T. ciliata* complex at the range-wide scale, which is complementary to and consolidates previous studies that examined the phylogeography of the *T. ciliata* complex using mtDNA and nuclear DNA markers. We analyzed 447 samples from 29 populations across the species’ distribution in China. Our specific conclusions are summarized as follows: (1) The overall haplotype diversity and genetic diversity were high, and their spatial distributions exhibited explicit geographic structure; (2) the haplotype diversity was larger in the western region than in the eastern region; (3) genetic differentiation among populations was intermediate (*Φ*_st_ = 42.84%), and significant effects of isolation by distance were present; and (4) the natural distribution of *T. ciliata* did not show apparent expansion, although a few local populations might expand after bottleneck effects. By synthesizing the overall results from organelle (mtDNA, cpDNA) and nuclear (SRAP, SSR, and ITS) genome markers, we suggest that a genetic conservation strategy in terms of the *T. ciliata* complex is appropriate. Attention to more populations in the western region than in the eastern region is suggested for the genetic conservation of the *T. cilata* complex. This also supports previous recommendations for the genetic conservation of this endangered species.

## Figures and Tables

**Table 1:** Localities and 447 individuals of 29 populations of the *T. ciliata* complex analyzed with cpDNA markers.

Code	Location	Sample Size	Longitude (E)	Latitude (N)	Elevation (m)
XJ	Xianju, Zhejiang	9	119.92	28.45	600
LL	Longlin, Guangxi	10	105.20	24.46	624
SM	Simao, Yunnan	11	100.58	22.46	1317
SC	Suichang, Zhejiang	12	119.25	28.59	510
CB	Chengbu, Hunan	23	111.28	27.14	737
XN	Xinning, Hunan	11	109.44	28.18	650
NP	Nanping, Fujian	9	118.10	26.38	800
JL	Jiulianshan, Jiangxi	18	114.47	24.54	610
DC	Dechang, Sichuan	29	102.32	26.40	1325
XY	Xingyi, Guizhou	15	104.54	25.06	1160
XL	Xilin, Guangxi	24	105.05	24.29	899
WM	Wangmo, Guizhou	30	106.05	25.10	500
YR	Yongren, Yunnan	27	101.32	25.01	1539
BS	Baoshan, Yunnan	11	99.06	25.04	1513
LD	Luodian, Guizhou	14	106.44	25.25	814
TL	Tianlin, Guangxi	26	106.13	24.17	792
CH	Ceheng, Guizhou	22	105.48	24.59	730
HD	Huidong, Sichuan	25	102.09	27.23	1862
JX	Jingxian, Anhui	15	118.24	30.41	366
HS	Huangshan, Anhui	6	118.08	30.16	499
WY	Wuyishan, Fujian	9	117.42	28.18	1200
JG	Jinggangshan, Jiangxi	5	114.17	26.44	400
XE	Xuanen, Hubei	9	109.28	30.17	632
GS	Guanshan, Jiangxi	11	115.26	27.19	337
PW	Puwen, Yunnan	21	101.04	22.23	890
MT	Maotoushan, Jiangxi	9	117.03	27.41	1000
YF	Yunfu, Guangdong	18	111.34	22.46	340
HP	Hupingshan, Hunan	3	111.41	29.01	436
LC	Lechang, Guangdong	15	113.20	25.07	359

**Table 2:** Tested cpDNA primer sequences in *T. ciliata* complex.

Primers	Sequence 5′-3′
*psb*A-*trn*H	GTTATGCATGAACGTAATGCTC
	CGCGCATGGTGGATTCACAATCC
*trn*T-*trn*L	CATTACAAATGCGATGCTCT
	TCTACCGATTTCGCCATATC
*trn*L-*trn*L	CGAAATCGGTAGACGCTACG
	GGGGATAGAGGGACTTGAAC
*trn*L-*trn*F	GGTTCAAGTCCCTCTATCCC
	ATTTGAACTGGTGACACGAG

**Table 3:** Haplotypes and haplotype diversity for the *psb*A-*trn*H fragment in each population of the *T. ciliata* complex.

Population	Observed Haplotype Diversity (*h*)	Nucleotide Diversity per Site (*π*)	Number of Haplotypes #	Types of Haplotypes
XJ	0.5679	0.0015	3 + 0	H1 H2 H3
LL	0.6600	0.0070	2 + 2	H4 H6 H47
SM	0.3140	0.0011	2 + 1	H9 H10 H47
SC	0.5139	0.0017	4 + 0	H1 H2 H3 H11
CB	0.8809	0.0084	6 + 5	H12 H13 H14 H16 H17 H19 H47
XN	0.9091	0.0712	5 + 6	H2 H17 H26 H27 H28 H47
NP	0.8025	0.0070	4 + 2	H1 H26 H28 H32 H47
JL	0.8765	0.0118	6 + 5	H1 H2 H27 H28 H32 H35 H47
DC	0.9489	0.0186	3 + 21	H5 H43 H44 H47
XY	0.5867	0.0046	4 + 1	H4 H6 H7 H8 H47
XL	0.7396	0.0088	4 + 4	H4 H6 H7 H15 H47
WM	0.9289	0.0321	7 + 15	H4 H6 H18 H20 H21 H22 H23 H47
YR	0.9163	0.0087	8 + 11	H9 H10 H24 H25 H29 H30 H31 H33 H47
BS	0.8926	0.0526	4 + 6	H10 H29 H30 H33 H47
LD	0.8980	0.0154	6 + 6	H6 H7 H18 H21 H22 H34 H47
TL	0.9586	0.0257	4 + 21	H22 H34 H36 H37 H47
CH	0.9504	0.0263	3 + 18	H8 H37 H38 H47
HD	0.9504	0.0214	1 + 22	H39 H47
JX	0.3467	0.0161	2 + 2	H2 H3 H47
HS	0.8333	0.1398	0 + 6	H47
WY	0.8148	0.0922	2 + 5	H40 H41 H47
JG	0.8000	0.0768	0 + 5	H47
XE	0.8889	0.0817	4 + 5	H1 H35 H41 H42 H47
GS	0.9091	0.1133	4 + 7	H40 H42 H45 H46 H47
PW	0.8753	0.0144	5 + 8	H9 H10 H25 H31 H33 H47
MT	0.8889	0.0164	2 + 7	H2 H45 H47
YF	0.0000	0.0000	1 + 0	H9
HP	0.4444	0.0027	2 + 0	H2 H3
LC	0.3378	0.0013	3 + 0	H2 H9 H46
Total	0.9767	0.0303	46 + 193	H1–H47

#: x + y means that there were x haplotypes whose abundances were not less than two and y haplotypes whose abundance was one in overall samples.

**Table 4:** Haplotypes and haplotype diversity for the *trn*L-*trnL* fragment in each population of the *T. ciliata* complex.

Population	Observed Haplotype Diversity (*h*)	Nucleotide Diversity per Site (*π*)	Number of Haplotypes #	Types of Haplotypes
XJ	0.0000	0.0000	1 + 0	H1
LL	0.8200	0.0031	4 + 3	H2 H3 H4 H5 H47
SM	0.7934	0.0076	4 + 2	H3 H4 H9 H11 H47
SC	0.0000	0.0000	1 + 0	H1
CB	0.3856	0.0008	2 + 0	H1 H13
XN	0.0000	0.0000	1 + 0	H1
NP	0.0000	0.0000	1 + 0	H1
JL	0.0000	0.0000	1 + 0	H1
DC	0.8252	0.0049	7 + 9	H16 H17 H19 H21 H22 H25 H28 H47
XY	0.9244	0.0084	7 + 7	H19 H31 H32 H34 H35 H39 H40 H47
XL	0.9375	0.0073	11 + 9	H6 H7 H8 H10 H12 H14 H31 H32 H34 H35 H39 H47
WM	0.9489	0.0085	12 + 13	H8 H15 H18 H20 H23 H24 H26 H31 H32 H34 H35 H39 H47
YR	0.9465	0.0076	14 + 8	H2 H5 H7 H8 H9 H11 H12 H14 H15 H27 H29 H32 H34 H35 H47
BS	0.9091	0.0066	8 + 3	H6 H7 H10 H14 H29 H31 H32 H35 H47
LD	0.7653	0.0028	6 + 1	H10 H15 H29 H32 H34 H35 H47
TL	0.7781	0.0032	9 + 3	H12 H23 H24 H26 H29 H30 H34 H35 H40 H47
CH	0.9215	0.0043	8 + 8	H18 H24 H30 H33 H34 H35 H36 H40 H47
HD	0.8928	0.0079	7 + 8	H16 H25 H28 H37 H38 H41 H42 H47
JX	0.1244	0.0005	2 + 0	H1 H9
HS	0.2778	0.0019	1 + 1	H1 H47
WY	0.4938	0.0086	2 + 2	H1 H36 H47
JG	0.7200	0.0154	3 + 1	H32 H35 H43 H47
XE	0.4938	0.0041	2 + 1	H1 H44 H47
GS	0.2975	0.0006	2 + 0	H1 H43
PW	0.9070	0.0077	9 + 8	H3 H4 H10 H12 H33 H34 H35 H45 H46 H47
MT	0.0000	0.0000	1 + 0	H1
YF	0.9321	0.0084	6 + 10	H8 H12 H27 H32 H35 H46 H47
HP	0.7778	0.0013	2 + 0	H1 H44
LC	0.2400	0.0027	3 + 0	H1 H9 H32
Total	0.8999	0.0189	46 + 97	H1–H47

#: x + y means that there were x haplotypes whose abundances were not less than two and y haplotypes whose abundance was one in overall samples.

**Table 5:** Analysis of molecular variance (AMOVA) using the *psb*A-*trn*H and *trn*L-*trn*L sequences and their concatenated sequences of the *T. ciliata* complex.

Marker	Source of Variation	d.f.	Sum of Squares	Variance Component	Percentage of Variance (%)	*Φ*_st_	*p*-Value
*psb*A-*trn*H+ *trn*L-*trn*L	Among population	28	2110.429	4.5357	42.84	0.4284	0.0000
	Within population	418	2529.542	6.0515	57.16		
	Total	446	4639.971	10.5873			
*psb*A-*trn*H	Among population	28	910.769	1.8021	26.55	0.2655	0.0000
	Within population	418	2083.932	4.9855	73.45		
	Total	446	2994.7	6.7876			
*trn*L-*trn*L	Among population	28	1202.878	2.7344	70.04	0.7004	0.0000
	Within population	418	488.911	1.1696	29.96		
	Total	446	1691.79	3.9040			

**Table 6:** The neutrality test and mismatch distribution analysis with the concatenated alignment sequences of the *psb*A-*trn*H and *trn*L-*trn*L fragments in 29 populations of the *T. ciliata* complex.

Population	Tajima’s D (*p*-Value)	Fu’s F (*p*-Value)	Mismatch Distribution				
			SSD (*p*-Value)	Rag (*p*-Value)	θ_0_	θ_1_	τ
XJ	−0.0638 (0.4)	−0.2393 (0.346)	0.0443 (0.15)	0.2677 (0.15)	0	3407.185	0.936
LL	−1.6802 (0.038)	1.6590 (0.811)	0.0394 (0.4)	0.0523 (0.8)	3.029	6914.97	1.172
SM	−1.5999 (0.044)	−0.5370 (0.129)	0.0219 (0.55)	0.0575 (0.45)	0	14.16	5.23
SC	0.6879 (0.796)	−1.0476 (0.106)	0.0079 (0.7)	0.0870 (0.85)	0	1.988	1.479
CB	0.4359 (0.726)	−2.046 (0.161)	0.0043 (0.85)	0.0168 (0.95)	0	6.392	3.711
XN	−0.6741 (0.244)	−1.441 (0.145)	0.2121 (0)	0.0519 (1)	3.025	3641.256	1.045
NP	0.7724 (0.794)	−0.8396 (0.24)	0.0149 (0.45)	0.0709 (0.95)	0.002	4.962	2.135
JL	−1.1649 (0.108)	−1.967 (0.179)	0.0541 (0.55)	0.1139 (0.4)	0	2.317	15.604
DC	−1.8304 (0.015)	−12.3959 (0)	0.0031 (0.6)	0.0098 (0.65)	3.7	6934.956	3.33
XY	−0.9887 (0.143)	0.2473 (0.572)	0.0055 (0.6)	0.2176 (0.6)	0.083	55.029	6.111
XL	−0.8636 (0.202)	0.4259 (0.616)	0.0038 (0.6)	0.0146 (0.85)	1.9	3414.978	3.029
WM	−2.4485 (0)	−3.9285 (0.092)	0.0423 (0)	0.0072 (1)	3.938	8040.089	6.094
YR	−0.3648 (0.424)	−14.8439 (0)	0.0009 (0.85)	0.0072 (0.95)	1.22	3045	6.717
BS	−2.0701 (0.001)	−0.3533 (0.356)	0.0367 (0.2)	0.0893 (0.05)	5.5	3414.978	7.451
LD	−0.8212 (0.217)	−4.4225 (0.018)	0.0085 (0.8)	0.0200 (0.7)	7.3	6854.957	2.77
TL	−1.6168 (0.028)	−15.1810 (0)	0.0421 (0)	0.0079 (1)	3.825	7174.983	5.74
CH	−1.0965 (0.129)	−10.8408 (0.001)	0.0086 (0.5)	0.0141 (0.55)	0	64.688	11.434
HD	−0.4642 (0.378)	−13.3933 (0)	0.0137 (0.05)	0.0089 (0.7)	5.4	3935.014	10.283
JX	−2.0409 (0)	6.0964 (0.989)	0.0195 (0.4)	0.2302 (0.55)	0	0.523	3
HS	−0.1419 (0.456)	1.2436 (0.423)	0.0583 (0.6)	0.0889 (0.9)	67.4	219.363	12.262
WY	−0.3811 (0.385)	3.2794 (0.923)	0.0613 (0.7)	0.0439 (0.85)	0.002	9.545	80.68
JG	0.8909 (0.805)	1.1153 (0.466)	0.0949 (0.35)	0.2200 (0.2)	72	157.478	43.17
XE	−0.2181 (0.446)	−0.4294 (0.26)	0.0371 (0.8)	0.0525 (0.75)	28.8	3515.191	0.895
GS	−1.1527 (0.114)	−0.6481 (0.222)	0.0562 (0.25)	0.0856 (0.25)	5.05	3474.992	1.227
PW	−1.7853 (0.018)	−2.7789 (0.092)	0.0029 (0.8)	0.0063 (0.95)	2.602	61.328	6.117
MT	−0.1726 (0.459)	−4.0639 (0.008)	0.0152 (0.3)	0.0478 (0.35)	1.9	80.986	5.871
YF	0 (1)	0 (1)	0.0686 (0)	0.0212 (0.9)	2.406	7354.997	2.4
HP	0 (1)	0.2007 (0.377)	0.1643 (0.3)	0.6667 (0.6)	0	6829.957	1.629
LC	−0.9481 (0.22)	−0.0064 (0.36)	0.3878 (0)	0.0863 (1)	1.143	3414.622	0

## Data Availability

All data sets used in this study are provided in Tables S1–S3 in the Supplementary Materials.

## Supplementary Materials

The following supporting information can be downloaded at: https://www.mdpi.com/article/10.3390/genes15030320/s1. Figure S1: Electrophoresis of PCR amplification products of two primer pairs (*psb*A-*trn*H and *trn*L-*trn*L); Figure S2: Sequencing peak maps checked using Chromatogram Explorer 3.2 for the *psb*A-*trn*H and *trn*L-*trn*L fragment amplifications; Table S1: Four hundred and forty-seven samples of concatenated alignment sequences of the *psb*A-*trn*H and *trn*L-*trn*L fragments; Table S2: Sequences of 239 haplotypes for the *psb*A-*trn*H fragment; Table S3: Sequences of 143 haplotypes for the *trn*L-*trn*L fragment.

## References

[1] Chen S.K., Li H., Chen B.Y. (1997). Meliaceae. Flora Reipublicae Popularis Sinicae.

[2] Ferreira D.M., Palma-Silva C., Neri J., Medeiros M.C., Pinange D.S., Benko-Iseppon A.M., Louzada R.B. (2021). Population genetic structure and species delimitation in the *Cryptanthus zonatus* complex (Bromeliaceae). Bot. J. Linn. Soc..

[3] Freeland J.R., Petersen S.D., Kirk H. (2011). Molecular Ecology.

[4] Xiao Y., Zhang X.X., Hu Y., Wang X., Li P., He Z.H., Lv Y.W., Chen X.Y., Hu X.S. (2023). Phylogeography of *Toona ciliata* (Meliaceae) complex in China inferred from cytonuclear markers. Genes.

[5] Edmonds J.M. (1993). The potential value of *Toona* species (Meliaceae) as multipurpose and plantation trees in Southeast Asia. Commonw. For. Rev..

[6] Edmonds J.M., Staniforth M. (1998). *Toona sinensis* (Meliaceae). Curtis’s Bot. Mag..

[7] Styles B.T. (1972). The flower biology of Meliaceae and its bearing on tree breeding. Silvae Genet..

[8] Liu J., Sun Z.X., Chen Y.T., Jiang J.M. (2012). Isolation and characterization of microsatellite loci from an endangered tree species, *Toona ciliata* var. *Pubescens*. Genet. Mol. Res..

[9] Cheng D.S., Cui T.L. (2010). Utilization value and cultivation techniques of *Toona sureni*. For. By-Prod. Spec. China.

[10] Liang R.L., Liao R.Y., Dai J. (2011). Endangered causes and protection strategy of *Toona ciliata*. Guangxi For. Sci..

[11] Wu J.Y., Cheng Y., Wang X.J., Chen R., Liu Q., Liu X.Y., Yin H.Y., Wu X.W. (2012). Softwood cutting propagation of *Toona ciliata* clones. Hunan For. Sci. Technol..

[12] Chen K. (2016). Technology of Tonna Tissue Cultivation Research. Master’s Thesis.

[13] Zhang H.B., Zhang G.Y., Huang G.Y., Wu D., Wang X.Y. (2016). Research on *Tonna ciliata* seedling and silviculture technology. Sci. Technol..

[14] Li P., Que Q.M., Wu L.Y., Zhu Q., Chen X.Y. (2017). Growth rhythms of *Toona ciliata* seedlings from different provenances. J. South China Agri. Univ..

[15] Wen W.H., Wu J.Y., Chen M.G., Chen J.F., Cheng Y., Wang X.J., Liu Q. (2012). Seedling growth performance of *Toona ciliata* elite trees progeny. China Agric. Sci. Bull..

[16] Liao D., Wu J., Shu Y., Cheng Y., Chen M., Chen J., Liu Q., Wu Z. (2017). Tissue culture of *Toona ciliata*. Agric. Sci. Technol..

[17] Lan J.H., Feng L.X. (2022). Study on cutting propagation technology of rare timber species *Toona ciliata* Roem. J. Guangxi Agric..

[18] Li P., Zhan X., Que Q.M., Qu W.T., Liu M.Q., Ouyang K.X., Li J.C., Deng X.M., Zhang J.J., Liao B.Y. (2015). Genetic diversity and population structure of *Toona ciliata* Roem. based on sequence-related amplified polymorphism (SRAP) markers. Forests.

[19] Xing P.Y., Liu T., Song Z.Q., Li X.F. (2016). Genetic diversity of *Toona sinensis* Roem in China revealed by ISSR and SRAP markers. Genet. Mol. Res..

[20] Zhan X., Li P., Hui W.K., Deng Y.W., Gan S.M., Sun Y., Zhao X.H., Chen X.Y., Deng X.M. (2019). Genetic diversity and population structure of *Toona ciliata* revealed by simple sequence repeat markers. Biotechnol. Biotechnol. Equip..

[21] Xiao Y., Wang X., He Z.H., Lv Y.W., Zhang C.H., Hu X.S. (2023). Assessing phylogenetic relationships among varieties of *Toona ciliata* in sympatry with chloroplast genomes. Ecol. Evol..

[22] Liu J., Jiang J.M., Chen Y.T. (2014). Genetic diversity of central and peripheral populations of *Toona ciliata* var. *pubescens*, an endangered tree species endemic to China. Genet. Mol. Res..

[23] Liu J., Chen Y.T., Jiang J.M., He G.P., Yu G.M. (2009). Study on population genetic structure in *Toona ciliata* var. *pubescens* with SSR. For. Res..

[24] Zhou W., Zhang X.X., Ren Y., Li P., Chen X.Y., Hu X.S. (2020). Mating system and population structure in the natural distribution of *Toona ciliata* (Meliaceae) in South China. Sci. Rep..

[25] Wang X., Xiao Y., He Z.H., Li L.L., Song H.Y., Zhang J.J., Cheng X., Chen X.Y., Li P., Hu X.S. (2022). A chromosome-level genome assembly of *Toona ciliata* (Meliaceae). Genome Biol. Evol..

[26] Wang X., Xiao Y., He Z.H., Li L.L., Lv Y.W., Hu X.S. (2022). Evolutionary divergence between *Toona ciliata* and *Toona sinensis* assayed with their whole genome sequences. Genes.

[27] Wolfe K.H., Li W.H., Sharp P.M. (1987). Rates of nucleotide substitution vary greatly among plant mitochondrial, chloroplast, and nuclear DNAs. Proc. Natl. Acad. Sci. USA.

[28] Hu Y., Wang X., Zhang X.X., Zhou W., Chen X.Y., Hu X.S. (2019). Advancing phylogeography with chloroplast DNA markers. Biodivers. Sci..

[29] Palmer J.D. (1985). Comparative organization of chloroplast genomes. Ann. Rev. Genet..

[30] Wright S. (1969). Evolution and the Genetics of Populations.

[31] Hu X.S., Ennos R.A. (1999). Impacts of seed and pollen flow on population differentiation for plant genomes with three contrasting modes of inheritance. Genetics.

[32] Doyle J.J., Doyle J.L. (1987). A rapid DNA isolation procedure for small quantities of fresh leaf material. Phytochemistry.

[33] Taberlet P., Fumagalli L., Wust-Saucy A.-G., Cosson J.-F. (1998). Comparative phylogeography and postglacial colonization routes in Europe. Mol. Ecol..

[34] Zhu S., Liu Q., Qiu S., Dai J., Gao X. (2022). DNA barcoding: An efficient technology to authenticate plant species of traditional Chinese medicine and recent advances. Chin. Med..

[35] Kumar S., Stecher G., Tamura K. (2016). MEGA7: Molecular evolutionary genetics analysis version 7.0. Mol. Biol. Evol..

[36] Librado P., Rozas J. (2009). DnaSP v5: A software for comprehensive analysis of DNA polymorphism data. Bioinformatics.

[37] Nei M. (1987). Molecular Evolutionary Genetics.

[38] Pons O., Petitt R.J. (1996). Measuring and testing genetic differentiation with ordered versus unordered alleles. Genetics.

[39] Grivet D., Petit R.J. (2002). Chloroplast DNA phylogeography of the hornbeam in Europe: Evidence for a bottleneck at the outset of postglacial colonization. Conserv. Genet..

[40] Wright S. (1951). The genetical structure of populations. Ann. Eugen..

[41] Excoffier L., Laval G., Schneider S. (2005). Arlequin (version 3.0): An integrated software package for population genetics data analysis. Evol. Bioinform..

[42] Meirmans P.G. (2006). Using the AMOVA framework to estimate a standardized genetic differentiation measure. Evolution.

[43] Rousset F. (1997). Genetic differentiation and estimation of gene flow from F-statistics under isolation by distance. Genetics.

[44] Mantel N. (1967). The detection of disease clustering and a generalized regression approach. Cancer Res..

[45] Alexander D.H., Novembre J., Lange K. (2009). Fast model-based estimation of ancestry in unrelated individuals. Genome Res..

[46] Tajima F. (1989). Statistical method for testing the neutral mutation hypothesis by DNA polymorphism. Genetics.

[47] Fu Y.X. (1997). Statistical tests of neutrality of mutations against population growth, hitchhiking and background selection. Genetics.

[48] Rogers A.R., Harpending H. (1992). Population growth makes waves in the distribution of pairwise genetic differences. Mol. Biol. Evol..

[49] Lu C.X., Zhang D.C., Wang D.B. (2001). Origin and taxonomic position of Chinese Toona (*Toona sinensis* (A. Juss.) Roem.). Bull. Bot. Res..

[50] Li S., Liu S.L., Pei S.Y., Ning M.M., Tang S.Q. (2020). Genetic diversity and population structure of *Camellia huana* (Theaceae), a limestone species with narrow geographic range, based on chloroplast DNA sequence and microsatellite markers. Plant Divers..

[51] Deng Y., Liu T., Xie Y., Wei Y., Xie Z., Shi Y., Deng X. (2020). High genetic diversity and low differentiation in *Michelia shiluensis*, an endangered *Magnolia* species in south China. Forests.

[52] Zhang G.L., Han Y., Wang H., Wang Z.Y., Xiao H.X., Sun M.Z. (2021). Phylogeography of *Iris loczyi* (Iridaceae) in Qinghai–Tibet Plateau revealed by chloroplast DNA and microsatellite markers. AoB Plants.

[53] Zhang X., Liu Y.H., Wang Y.H., Shen S.K. (2020). Genetic Diversity and Population Structure of *Rhododendron rex* Subsp. *rex* Inferred from Microsatellite Markers and Chloroplast DNA Sequences. Plants.

[54] Dutech C., Maggia L., Joly H.I. (2000). Chloroplast diversity in *Vouacapoua americana* (Caesalpiniaceae), a neotropical forest tree. Mol. Ecol..

[55] Fineschi S., Salvini D., Taurchini D., Carnevale S., Vendramin G.G. (2003). Chloroplast DNA variation of *Tilia cordata* (Tiliaceae). Can. J. For. Res..

[56] Hamrick J.L., Murawski D.A. (1990). The breeding structure of tropical tree populations. Plant Species Biol..

[57] Hu X.S., Yeh F.C., Hu Y., Deng L.T., Ennos R.A., Chen X.Y. (2017). High mutation rates explain low population genetic divergence at copy-number-variable loci in *Homo sapiens*. Sci. Rep..

[58] Hamrick J.L., Godt M.J.W., Sherman-Broyles S.L. (1992). Factors influencing levels of genetic diversity in woody plant species. New For..

[59] Zhu Q., Liao B.Y., Li P., Li J.C., Deng X.M., Hu X.S., Chen X.Y. (2017). Phylogeographic pattern suggests a major eastward dispersal in the distribution of *Machilus pauhoi* in South China. PLoS ONE.

[60] Hu Y. (2019). Phylogeographic Structure of *Toona ciliata* (Meliaceae) in China Inferred from cpDNA Markers and Ecological Niche Model Analyses. Master’s Thesis.

[61] Zhang C.H., He J., Sun Y.Y., Li K. (2018). Prediction of distributional change of *Toona ciliata* var. *ciliata* and application in regionalization of introduction based on MaxEnt. J. Yunnan Univ..

[62] Zhang C.H., He J., Sun Y.Y., Li K. (2018). Distributional change in suitable areas for *T. ciliata* var. *pubescens* based on MaxEnt. For. Res..

