# Supplementary material for: Population Structure and Genetic Diversity of the Toona ciliata (Meliaceae) Complex Assayed with Chloroplast DNA Markers
Source: Genes (Basel). 2024 Feb 28;15(3):320. doi: 10.3390/genes15030320 (PMC10970150; doi:10.3390/genes15030320)
Supplement: Supplementary file 1 [file genes-15-00320-s001.zip › genes-2866691-supplementary/Figure S1 and S2.pdf]

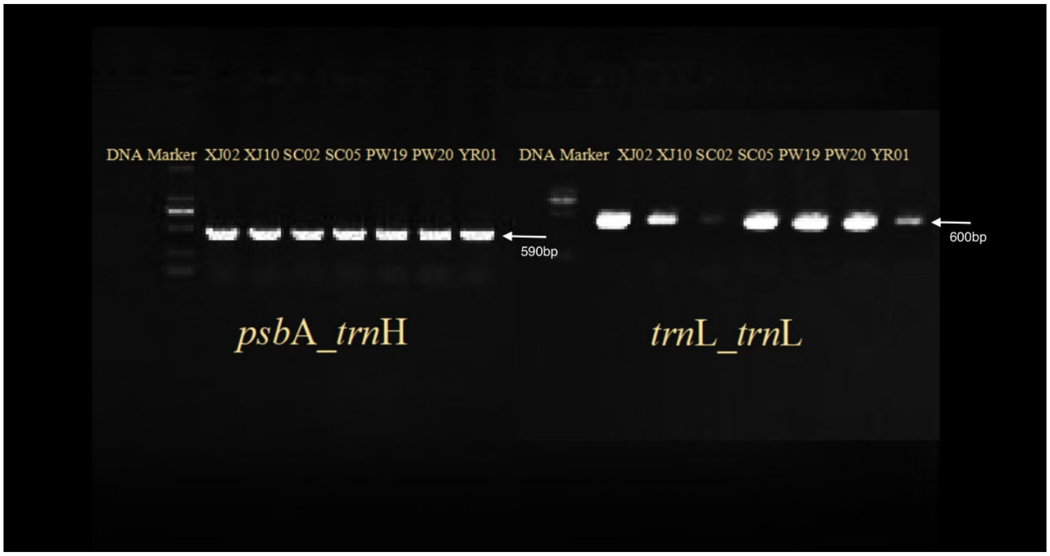

Figure S1. Electrophoresis of PCR amplification products of two primer pairs (*psb*A-*trn*H and *trn*L-*trn*L). Seven samples from four populations (XJ, SC, PW, and YR) were marked on the top line. Band sizes were indicated for amplifications of each primer pair.

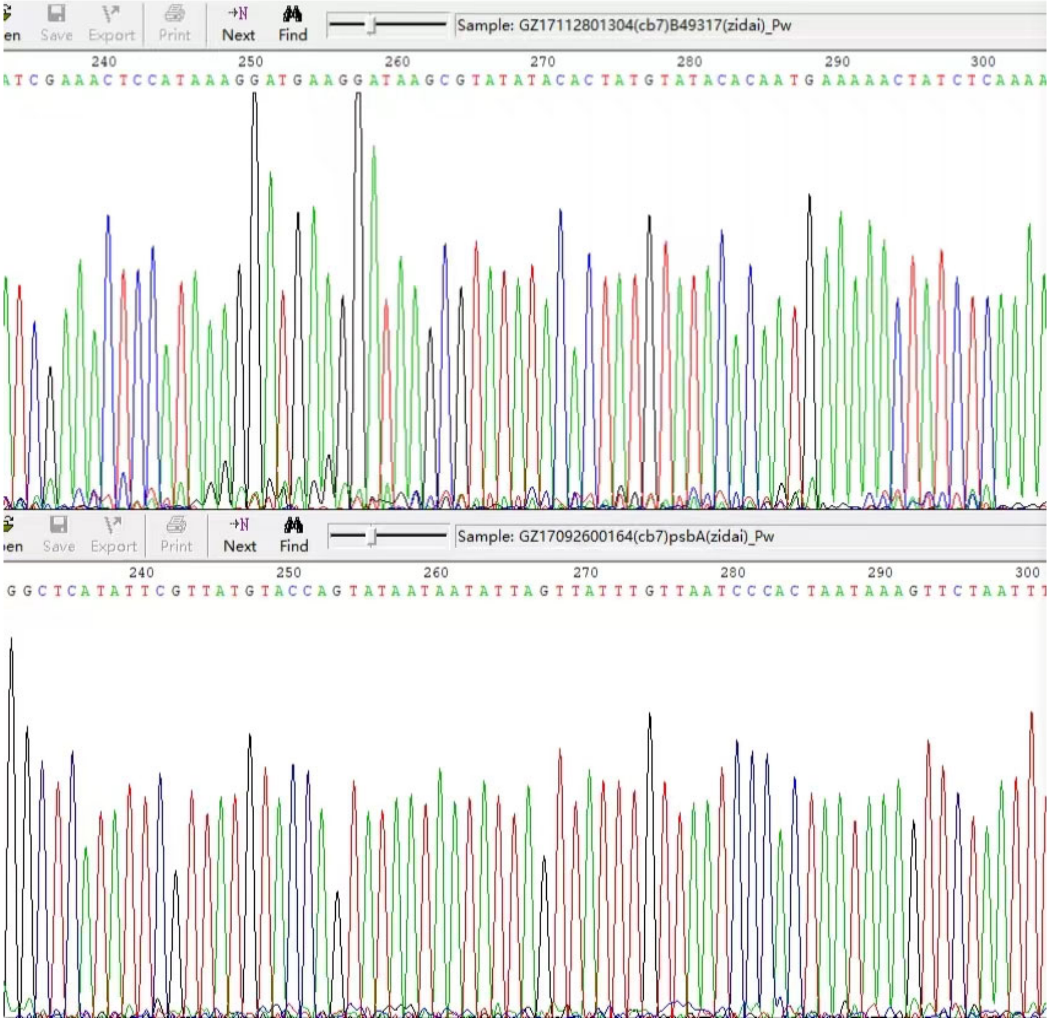

Figure S2. Sequencing peak maps were checked using Chromatogram Explorer 3.2. for *psb*A-*trn*H and *trn*L-*trn*L fragment amplifications. Bases A, T, G and C were indicated in different colors. The top figure was for PCR amplification of *psb*A-*trn*H and the bottom was for *trn*L-*trn*L of individual CB07 from Chengbu, Huanan Province.
